# 

**Published:** 1896-11

**Authors:** 


Vol XVII. NOVEMBER, 1896. No. 11.

PUBLISHED MONTHLY AT $3 A YEAR. SINGLE COPIES, 30 CENTS.

### THE JOURNAL OF COMPARATIVE MEDICINE AND VETERINARY ARCHIVES

EDITED AND PUBLISHED BY

RUSH SHIPPEN HUIDEKOPER, Veterinarian (Alfort),

W. HORACE HOSKINS, D.V.S.,

H. D. GILL, V.S.

### UNITED STATES VETERINARY MEDICAL ASSOCIATION.

**Proceedings of the Thirty-third Annual Meeting held at Buffalo, N. Y., September, 1896. (Continued.)**

Reports of State Secretaries—*continued* . . . 33

Report of the Committee on Publication . . . 49

Report of the Committee on Diseases . . . 55

ALL COMMUNICATIONS AND PAYMENTS SHOULD BE ADDRESSED TO

**Dr. W. Horace Hoskins, Managing Editor, 3452 Ludlow St., Philadelphia, Pa.**

Copies may be had on order of all news-dealers. In London of Baillière, Tindall & Cox.

